# Oblique incisions in hamstring tendon harvesting reduce iatrogenic injuries to the infrapatellar branch of the saphenous nerve

**DOI:** 10.1007/s00167-017-4590-y

**Published:** 2017-06-01

**Authors:** Brandon Michael Henry, Krzysztof A. Tomaszewski, Przemysław A. Pękala, Matthew J. Graves, Jakub R. Pękala, Beatrice Sanna, Ewa Mizia

**Affiliations:** 1 International Evidence-Based Anatomy Working Group, 12 Kopernika St, 31–034 Krakow, Poland; 2 Department of Anatomy, Jagiellonian University Medical College, 12 Kopernika St, 31–034 Krakow, Poland; 3 Faculty of Medicine and Surgery, University of Cagliari, S.S. 554 Bivio Sestu, 09042 Monserrato, CA, Sardinia Italy

**Keywords:** ACL, Anterior cruciate ligament reconstruction, Cadaveric simulation, Hamstrings, Iatrogenic injury, Infrapatellar branch of the saphenous nerve, IPBSN, Tendon harvesting

## Abstract

**Purpose:**

Iatrogenic injury to the infrapatellar branch of the saphenous nerve (IPBSN) is associated with many surgical interventions to the medial aspect of the knee, such as anterior cruciate ligament (ACL) reconstruction. Different types of surgical incisions during hamstring tendon harvesting for ACL reconstruction are related to a variable risk of IPBSN injury. This study aimed to evaluate the risk of iatrogenic IPBSN injury during hamstring tendon harvesting for ACL reconstruction with different incision techniques over the pes anserinus.

**Methods:**

This study was performed on 100 cadavers. Vertical, horizontal, or oblique incisions were simulated on each cadaveric limb to determine the incidence of iatrogenic IPBSN injury.

**Results:**

The vertical incision caused the IPBSN injury during hamstring tendon harvesting in 101 (64.7%), the horizontal incision in 78 (50.0%), and the oblique incision in 43 (27.6%) examined lower limbs. The calculated odds ratios (OR) for risk of injury in vertical versus horizontal and horizontal versus oblique incisions were 2.4 (95% CI 1.5–3.6) and 1.8 (95% 1.2–2.8), respectively.

**Conclusions:**

The vertical incision technique over the pes anserinus should be avoided during hamstring tendon harvesting for ACL reconstruction. The adoption of an oblique incision, with the shortest possible length, will allow for the safest procedure possible, thus minimizing the risk of iatrogenic IPBSN injury, and improving patient outcomes and postoperative quality-of-life.

## Introduction

**Fig. 1:**
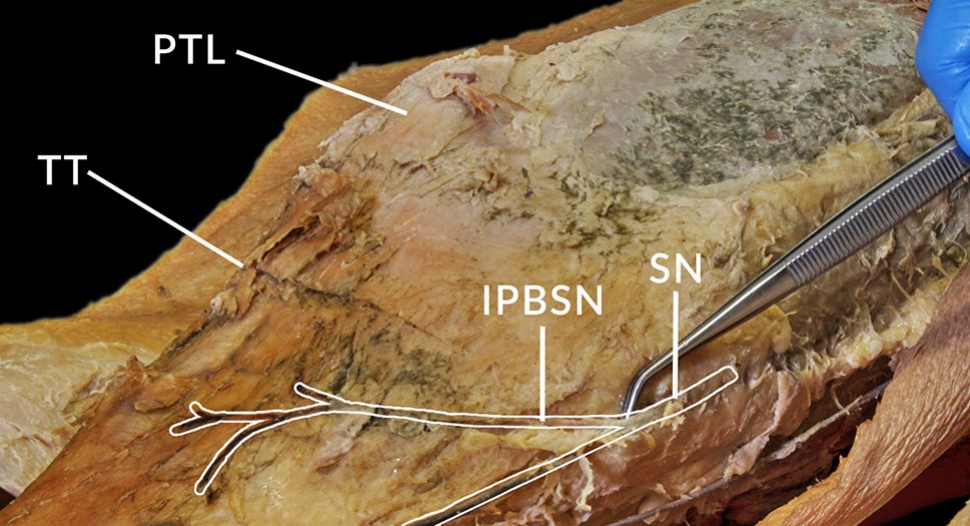
A dissected right limb showing the course of the IPBSN branching off the saphenous nerve. *IPBSN* infrapatellar branch of the saphenous nerve; *PTL* patella; *SN* saphenous nerve; and *TT* tibial tuberosity

The infrapatellar branch of the saphenous nerve (IPBSN) is a cutaneous nerve of the lower limb which arises distal to the adductor or subsartorial canal (Fig. 1) [10]. The nerve pierces the fascia lata running in a superficial course and innervating the skin over the anterior aspect of the knee, anterolateral aspect of the proximal lower limb, and anteroinferior aspect of the knee joint capsule [12, 14]. The anatomy of the IPBSN varies among individuals and can even vary in both limbs of the same individual [12]. Iatrogenic injury of the IPBSN is associated with many surgical interventions involving the medial aspect of the knee resulting in sensory symptoms, neuropathic pain, and painful neuroma [1, 2, 5, 6, 8, 12, 14, 18, 19, 27, 30].

**Fig. 2:**
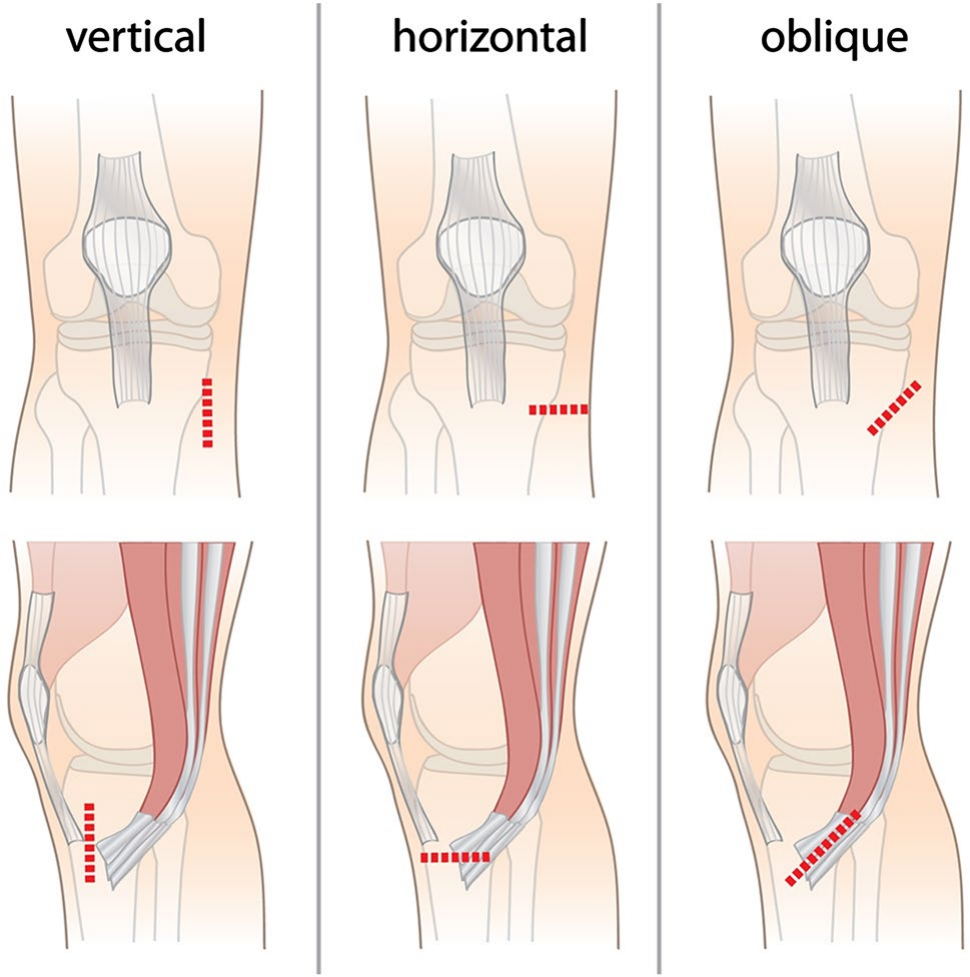
Different techniques of incision during hamstring tendon harvesting (*vertical*, *horizontal*, and *oblique*). The incisions are indicated with *red dashed lines* on the anterior view of the right knee

Anterior cruciate ligament (ACL) reconstruction is one of the most common procedures which may result in IPBSN injury. The procedure is often performed using either a hamstring tendon or a patellar tendon autograft [30]. Although hamstring tendon harvesting carries less risk of injury to the IPBSN between the two approaches, postoperative complications are not uncommon and vary between 12 and 88% [28]. Proximity of the hamstring tendons (semitendinosus and gracilis tendons) at the pes anserinus area to the IPBSN predisposes the nerve to damage during harvesting [25]. Specifically, different types of surgical incisions (Fig. 2) at the area for hamstring tendon harvesting are related to variable risk of injury to the IPBSN [28]. Vertical, horizontal, and even oblique incision methods have been proposed to reduce the risk of nerve injury based on anatomical findings of the IPBSN distribution [16, 19, 22, 24]. Nevertheless, no consensus has been reached regarding the optimal incision method at the pes anserinus area for hamstring tendon harvest. Various studies have attempted to identify the influence of incision orientation during the procedure and the incidence of postoperative IPBSN injury [15, 16, 21, 23, 25, 28]. Results of these studies may be incomparable due to the different sizes of incisions and the distance of the incision from major anatomical landmarks in the region such as the tibial tuberosity or the medial joint line. Some studies have even attempted to identify safe and/or risk zones at the pes anserinus area for hamstring tendon autograft harvesting [6, 14, 18, 19, 26, 29]. Safe zones, however, are quite challenging to define due to the significantly high variation in the topographical anatomy of the IPBSN [5, 12–14, 30]. Additionally, the number of clinically comparative studies are minimal, with the few published having small sample sizes and largely comparing only two types of incision method.

The purpose of this study was to simulate and evaluate the risk of iatrogenic injury to the nerve during hamstring tendon graft harvesting to determine the safest incision type. Additionally, the aim of this study was to provide surgeons with valuable information regarding the estimated risk of nerve injury in relation to orientation of surgical incisions. It was hypothesized that the oblique incision type is the safest for hamstring tendon graft harvesting [24]. However, previously conducted studies assessing this topic have been unable to reach uniform consensus largely due to poor methodology, making it impossible to draw definitive conclusions from existing literature.

In this study, all three incisions were performed on each knee. Such an approach was designed specifically for a direct and reliable comparison. In cases in which the incision did not cross the IPBSN, the distance between the nerve and the incision line was measured. Such knowledge may help surgeons to better understand how close the incision line is located to the IPBSN. Moreover, to the best of the authors’ knowledge, this study is the largest cadaveric investigation to date comparing the incidence of the IPBSN injury in relation to three incision types used for hamstring tendon harvesting.

## Materials and methods

### Cadaveric study

The study was performed on 100 fresh-frozen cadavers (48 females and 52 males, *n* = 200 lower limbs, 100 right and 100 left limbs). The mean age of donors was 62.3 ± 15.6 years (only adult cadavers were included in this study). Limbs with pathology (ex. congenital malformations) or history of trauma were excluded from the study. All analyses were performed on cadavers with clearly visible and identifiable nerves. Cadavers without nerves were excluded from the analysis.

Three different techniques of incision during hamstring tendon harvesting (vertical, horizontal, or oblique) were simulated on each lower limb, 3 cm over the pes anserinus region (Fig. 2). Incisions were conducted with the knee in a position of flexion and the hip in a position of external rotation. Subsequently, a second incision was made on the medial aspect of the thigh to locate and identify the saphenous nerve. This secondary incision was extended inferiorly along the sartorius muscle with further extension to the tibial tuberosity. A layer-by-layer blunt dissection technique was implemented to ascertain if the simulated incision resulted in iatrogenic injury of the IPBSN. The incidence of iatrogenic injury to the IPBSN for each type of incision was determined. If the incision did not cross the IPBSN, the distance between the nerve and the incision line was measured using an electronic caliper (Mitutoyo, Japan). Each measurement was performed twice using the same caliper (every time by the same two, equally experienced anatomists)—and the mean of both measurements was reported. All dissections were performed by experienced anatomists, with over 10 years of experience in cadaveric dissection.

### Statistical analysis

The statistical analysis was performed using MedCalc version 16.8. Data were analysed using elements of descriptive statistics (mean, SD, percentage distribution). For comparisons of the proportions between subgroups, the Chi-squared test was employed. Differences between the means of two groups were calculated using the student’s *t* test. To directly compare the different incision types, odds ratios (OR) were calculated. For all analysis, *p* < 0.05 was deemed statistically significant. Additionally the interclass correlation (ICC) was calculated for the measurements performed.

Sample size has been calculated using the following information (for calculating differences in proportions) [31]—the estimated incidence of iatrogenic IPBSN injury using the different incision techniques was assumed, based on the literature data, to fit within the 15–35% range; alpha value of 0.05; and beta value of 0.10. Thus, to obtain a power of 90% it was necessary to include a total of 192 lower limbs. It was decided to include 200 lower limbs to account for unexpected deviations from the assumed range of iatrogenic IPBSN injury incidence.

## Results

### Measurement of interclass correlation

Mean measurements closely approximated what was truly measured, as the ICC for the measurements was 97.3%.

### Prevalence of the IPBSN

The prevalence of the IPBSN among the dissected lower limbs (*n* = 200) was 78.0%. The nerve was slightly more common on the right (79 lower limbs, 79.0%) than on the left side (77 lower limbs, 77.0%), and among females (75 lower limbs, 78.1%), than males (81 lower limbs, 77.9%), albeit all not significantly (all *p* values non-significant). For all subsequent statistical analyses, *n* = 156 limbs (male right = 42, male left = 39, female right = 37, female left = 38). The vertical incision led to IPBSN injury during hamstring tendon harvesting in 101 (64.7%), the horizontal incision in 78 (50.0%), and the oblique incision in 43 (27.6%) examined lower limbs.

### Incidence of simulated iatrogenic injury to the IPBSN

The incidence of iatrogenic injury to the IPBSN with different types of simulated incisions revealed that the vertical incision was associated with the greatest risk of injury during simulated hamstring tendon harvesting (101 lower limbs, 64.7%) as compared to horizontal (78 lower limbs, 50.0%; *p* = 0.009), and oblique (43 lower limbs, 27.6%, *p* < 0.001). No significant differences were found for any incision type between right and left limbs or between males and females (all *p* values non-significant). Detailed data on iatrogenic injury to the nerve with different simulated incision techniques are presented in Table 1. Odds ratios comparing the vertical and horizontal incisions versus oblique incision were statistically significant and showed that the oblique incision should be performed over the vertical and horizontal procedures (Table 2).

**Table 1:** Incidence of iatrogenic IPBSN injury with different surgical incisions during hamstring tendon harvesting (if incision did cross the IPBSN)

Incision orientation	Laterality of the lower limb	Overall *N* (%)	Male	Female
Vertical	Both	101 (64.7)	49 (60.5)	52 (69.3)
	Right	49 (62.0)	24 (57.1)	25 (67.6)
	Left	52 (67.5)	25 (64.1)	27 (71.1)
Horizontal	Both	78 (50.0)	40 (49.4)	38 (50.7)
	Right	40 (50.6)	19 (45.2)	21 (56.8)
	Left	38 (49.4)	21 (52.9)	17 (44.7)
Oblique	Both	43 (27.6)	26 (32.1)	17 (22.7)
	Right	21 (26.6)	12 (28.6)	9 (24.3)
	Left	22 (28.6)	14 (35. 9)	8 (21.1)

Total *N* = 156 (male right = 42, male left = 39, female right = 37, female left = 38); *IPBSN* infrapatellar branch of the saphenous nerve, *N* number of injured limbs

**Table 2:** Odds ratios for IPBSN injury—comparison of different incision types for cadaveric

Incision types	Cadaveric: OR (95% CI)
Vertical versus oblique	2.4 (1.5–3.6)
Vertical versus horizontal	1.3 (0.9–1.9)
Horizontal versus oblique	1.8 (1.2–2.8)

### Distance between the IPBSN and the different incision lines

In cases where the incision did not cross the IPBSN, the mean distances between the incision line and the IPBSN were 8.6 ± 2.8, 8.7 ± 3.0, and 8.2 ± 2.8 mm for vertical, horizontal, and oblique incisions, respectively. No statistically significant differences were found for any incision type between right and left limbs or between males and females. Detailed data on the mean distances are presented in Table 3.

**Table 3:** Distance between the IPBSN and the different incision lines during hamstring tendon harvesting (if the incision did not cross the IPBSN)

Incision orientation	Laterality of the lower limb	Distance, mean ± SD (mm)	Male	Female
Vertical	Both	8.6 ± 2.8	8.6 ± 2.7	8.5 ± 2.9
	Right	8.1 ± 2.6	8.2 ± 2.3	8.0 ± 3.1
	Left	9.0 ± 2.9	9.0 ± 3.1	9.1 ± 2.8
Horizontal	Both	8.7 ± 3.0	9.3 ± 3.1	8.0 ± 2.8
	Right	9.2 ± 3.3	9.6 ± 3.5	8.6 ± 3.1
	Left	8.2 ± 2.6	8.9 ± 2.5	7.6 ± 2.6
Oblique	Both	8.2 ± 2.8	8.4 ± 3.3	8.0 ± 2.4
	Right	8.3 ± 3.2	8.5 ± 3.8	8.1 ± 2.5
	Left	8.1 ± 2.4	8.3 ± 2.6	7.9 ± 2.3

## Discussion

This study aimed to assess the risk of iatrogenic injury to the IPBSN associated with three different incision techniques utilized during hamstring tendon harvesting during ACL reconstruction.

Our cadaveric investigation demonstrated that a vertical incision during hamstring tendon harvesting should be avoided, as it is associated with the highest rate of iatrogenic injury to the IPBSN among all analysed incision types (64.7%). Therefore, we recommend the utilization of the oblique incision during hamstring tendon harvesting [22]. Horizontal incisions were also associated with increased risk of injury when compared to oblique with 50% of these incisions inciting IPBSN injury. Surgeons who opt for the riskier vertical [OR: 2.35 (95% CI 1.54–3.58)] or horizontal [OR 1.81 (1.18–2.80)] incisions are subjecting their patients to increased and unnecessary risks. The oblique incision is parallel to the course of IPBSN fibres, allowing for visualization of the nerve when performing the incision, and thus decreasing the risk of iatrogenic damage [6]. Moreover, the authors of this study encourage orthopaedic surgeons to use blunt dissection techniques and to exercise caution during wound closure [18].

In cases of no injury observed, the average distances from the incision line to the IPBSN were relatively small for all incision types (8.2–8.7 mm) as demonstrated in our cadaveric investigation. This finding advocates using the shortest incision possible during hamstring tendon harvesting and explains the very high IPBSN injury rate (84.0%) seen in the clinical study by Kjaergaard et al. [15]. As such, the length of incision appears equally important in preventing iatrogenic injury to the IPBSN, as is the incision orientation.

A recent study using Computer Assisted Surgical Anatomy Mapping (CASAM) showed that the course of the IPBSN is highly variable, with numerous small terminal branches covering almost the whole anteromedial aspect of the knee that cannot be revealed during USG examination [14]. This is an important factor to consider when addressing post-procedural complications, as painful neuromas have been known to develop in cases when non-visible terminal branches of the IPBSN get transected [11]. Various safe zones, places where the risk of finding IPBSN nerve fibres are minimal, have been proposed by some researchers [6, 14, 18]. The zones least vulnerable to injury were near the medial border of the patella and the patellar ligament (1.0–3.1 cm from medial border of the patellar ligament) [6, 14, 18]. Further validation, however, is needed, with both anatomical and clinical studies to determine the practical utility of these safe zones.

The anterior cruciate ligament is the most commonly injured knee ligament, especially among athletes and sport trauma victims [3, 7]. Among the most commonly used grafts (patellar tendon graft, hamstring tendon graft, and allograft), the hamstring tendon autograft remains popular because of the decreased postoperative incidence of patellofemoral crepitation, kneeling pain, and extension loss [9]. During medial hamstring tendon harvesting, the incision is made over the pes anserinus, at an approximate distance of 2.5–4 cm from the tibial tuberosity [28].

The IPBSN usually innervates the skin on the anteromedial aspect of the knee between the patellar apex and tibial tuberosity [28]. Damage to the IPBSN has been reported after ACL reconstruction using hamstring tendon autografts [8, 19, 30]. Several authors have suggested that iatrogenic injury to the nerve may occur during skin incision, subcutaneous dissection, portal placement, tendon harvesting, or tibial tunnel drilling [6, 19, 20, 26]. The incidence of nerve injury during grafting increases when both the semitendinosus and gracilis tendons are used as grafts compared to the semitendinosus alone [19].

Symptoms of IPBSN injury may include anaesthesia, hypoesthesia, dysesthesia, or paraesthesia on the anteroinferior, anteromedial, and even anterolateral knee [5, 14, 30]. Neuropathic pain and painful neuroma have also been reported in patients with IPBSN injury [1, 2]. In some cases, reflex sympathetic dystrophy has been described as a result of IPBSN injury after arthroscopic surgery [18]. Although the IPBSN is a purely sensory nerve, complications due to its injury often cause decreased patient satisfaction [28].

A review of the current literature does not advocate a standard incision method for hamstring tendon harvesting, for each type of incision has its own advantage and appeal to surgeons. An oblique incision at the pes anserinus provides a good exposure of the tendons (semitendinosus and/or gracilis) for harvesting and does not restrict the surgeon to the horizontal plane when determining the starting point for tibial tunnel drilling [4, 15, 17]. Vertical incisions enable alteration in the position of the guide around a transversal axis during tibial tunnel drilling by providing more space for inclination changes of the tunnel [15]. In horizontal incisions, the sufficiently distal location of the scar would theoretically avoid any direct pressure with kneeling or associated complications and has a better cosmetic outcome [23]. Nonetheless, numerous studies [15, 16, 21, 25, 28] have attempted to study the possible correlation between the different types of incision and the risk of IPBSN injury during the procedure.

Noteworthy is the fact that during movements of the knee joint, the position of the IPBSN changes. Extension of the knee joint and placement of the hip joint in its classical anatomical position causes a shift of the IPBSN in the proximal direction, increasing nerve tension, making it more difficult to visualize, and therefore more vulnerable to injury [20, 21]. As such, the above mentioned position should be avoided during hamstring tendon harvesting.

The authors of the present study opted to use cadavers and dissection methods, believing that it is the “gold standard” for nerve course visualization and the ideal reference method to investigate the detailed anatomy of the IPBSN [5].

A significant limitation of this study was the inability to visualize the tiniest IPBSN branches during dissection. To minimize this risk, a magnifying glass and microdissection tools were employed during our cadaveric investigation. We do acknowledge that this was likely the main contributing factor to the low overall prevalence of the IPBSN in the present study. We believe that the true prevalence rate in the Polish population is likely higher as is reported in other works. We hypothesize that there were presumably smaller direct branches from either the femoral or saphenous nerves supplying the territory of the IPBSN when the main IPBSN was noted to be absent. To visualize such small terminal branches would require the employment of histological techniques and should be explored in the future. As the prevalence of the IPBSN has been reported to be quite high in other studies across various populations, surgeons should operate under the assumption that it is always present. Nonetheless, future studies should investigate the prevalence of the IPBSN in the Polish and other European populations, and when absent, what supplies innervation to the IPBSN territory. It is unclear in prior investigations as to whether studies only included specimens with intact IPBSNs or that the studies did truly have a higher prevalence. Additionally, due to the underlying methodology of the study, sensory changes after incision could not be assessed at any point.

## Conclusions

The results of our cadaveric simulation revealed that a vertical incision technique over the pes anserinus region during hamstring tendon harvesting for ACL reconstruction should be avoided to reduce the risk of IPBSN injury and subsequent sensory disturbances. An oblique incision of the shortest length possible is the optimal choice based on our results allowing for minimal risk of the iatrogenic nerve injury.

## Abbreviations

IPBSN: Infrapatellar branch of the saphenous nerve
ACL: Anterior cruciate ligament
OR: Odds ratio
PTL: Patella
TT: Tibial tuberosity
SN: Saphenous nerve
